# Using weather radar to monitor the number, timing and directions of flying-foxes emerging from their roosts

**DOI:** 10.1038/s41598-019-46549-2

**Published:** 2019-07-15

**Authors:** Jessica Meade, Rodney van der Ree, Phillip M. Stepanian, David A. Westcott, Justin A. Welbergen

**Affiliations:** 1 Hawkesbury Institute for the Environment, Western Sydney University, Richmond, NSW 2751 Australia; 2 School of BioSciences, The University of Melbourne, Parkville Victoria, 3010 Australia; 3 Ecology and Infrastructure International, Wantirna Victoria, 3152 Australia; 4 Plains Institute, University of Oklahoma, Norman, Oklahoma 73019 USA; 5 Commonwealth Scientific and Industrial Research Organisation (CSIRO), Land and Water, 67 Maunds St., Atherton, Queensland 4883 Australia

**Keywords:** Conservation biology, Behavioural ecology

## Abstract

Knowledge of species’ population trends is crucial when planning for conservation and management; however, this information can be difficult to obtain for extremely mobile species such as flying-foxes (*Pteropus* spp.; Chiroptera, Pteropodidae). In mainland Australia, flying-foxes are of particular management concern due their involvement in human-wildlife conflict, and their role as vectors of zoonotic diseases; and two species, the grey-headed flying-fox (*Pteropus poliocephalus*) and the spectacled flying-fox (*P. conspicillatus*), are currently threatened with extinction. Here we demonstrate that archival weather radar data over a period of ten years can be used to monitor a large colony of grey-headed flying-foxes near Melbourne. We show that radar estimates of colony size closely match those derived from traditional counting methods. Moreover, we show that radar data can be used to determine the timing and departure direction of flying-foxes emerging from the roost. Finally, we show that radar observations of flying-foxes can be used to identify signals of important ecological events, such as mass flowering and extreme heat events, and can inform human activities, e.g. the safe operation of airports and windfarms. As such, radar represents an extremely promising tool for the conservation and management of vulnerable flying-fox populations and for managing human interactions with these ecologically-important mammals.

## Introduction

Monitoring population trends is a fundamental component of species conservation and management, and is of growing importance as human impacts increase the necessity for conservation management of wild populations^1^. Knowledge of species abundance, distribution, and dynamics is crucial for conservation planning, and also allows for the assessment of the effectiveness of management activities and the impacts of random, catastrophic events^2,3^. Highly social and highly volant animals, such as some birds and bats, can be difficult to monitor using traditional methods, as they often occur in large numbers, perhaps fleetingly, in multiple locations simultaneously, and because their mobility allows them to rapidly cross geopolitical boundaries^4^.

Radars provide one solution to monitoring such species. Flying animals have been detected on radar scans ever since they were first used during the second world war^5^. In recent years the field of aeroecology has grown rapidly with improvements in the technologies being used and in the distribution of installations. Weather radar archives are now used widely to monitor free-ranging volant animals^4^, and can provide information on landscape scale movements of a variety of organisms^6^. Ground-based weather surveillance radar data have been of importance in bat research for almost 50 years^4,7–11^, but few studies have used radar to monitor bat populations (e.g.^12,13^), and only very recently has radar been used to quantify bat numbers^14^.

Old world flying-foxes (Pteropodidae) are an example of highly mobile species that can occur in substantial numbers and, despite their habit of roosting in large colonies, are difficult to monitor due to their extreme mobility and often inaccessible roosting sites^1^. In Australia, two of the four mainland species of flying-fox (grey-headed flying-fox *Pteropus poliocephalus* and spectacled flying-fox *P. conspicillatus*) are listed as threatened with extinction under federal legislation, and *P. poliocephalus* is also on the IUCN red list as vulnerable (IUCN 2018). These animals provide vital ecosystem services in terms of pollination and seed dispersal^15,16^, but are threatened by loss of foraging and roosting habitat^17^, direct killing and harassment of animals in orchards^18^, and mass die-off events when temperatures exceed 42 °C^19^. Australian flying-foxes have also been linked to several emerging zoonotic diseases^20–22^, which can be a cause of significant public concern. Thus data on flying-fox distribution and abundance are needed to provide insight into population trends and to inform management responses. In 2013 the National Flying-Fox Monitoring Program was implemented by Australia’s national science agency (CSIRO) with support from state and local governments, with the aim of counting the flying-foxes in all known daytime roosts of *P. poliocephalus* and *P. conspicillatus* across the species’ ranges once per quarter^23^. This program, like other flying-fox monitoring programs (e.g. https://megabatcount.wordpress.com), involves traditional methods of counting animals in colonies via fly-out and static (or walk-through) counts^1,24^. Both methods have several sources of error^1,25–27^ and are time consuming and costly.

Here, we propose that radar data can be used as a supplementary method for monitoring flying-foxes. In this paper we use the Yarra Bend colony of grey-headed flying-foxes near Melbourne as a case study demonstrating the potential use of archival weather radar data to monitor flying-fox population dynamics. This colony is ideal for this purpose as it has been monitored at fortnightly to monthly intervals via traditional counting methods since 2003^24^ (van der Ree unpub. data), and it is close to the Melbourne radar station. We show that emergence counts, including timing and direction of emergence can be estimated retrospectively, at a daily resolution. These data can be used to estimate local flying-fox abundance, can inform local airports in relation to the risk of flying-fox strike, and generates exciting new opportunities for investigating the drivers of flying-fox movements and population redistribution at a landscape scale.

## Results

### Long term flying-fox counts

**Figure 1:**
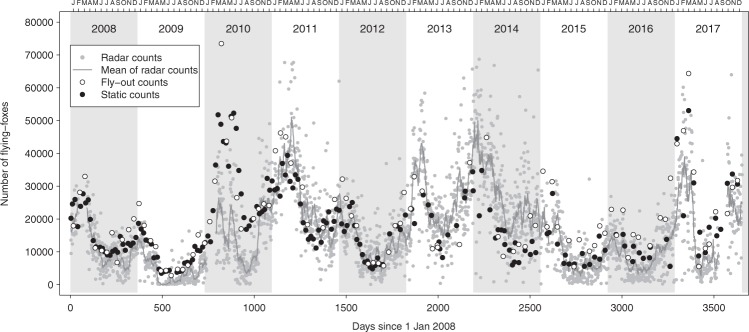
Radar-based and ground-based estimates of the numbers of flying-foxes at the Yarra Bend roost between 2008 and 2017. Radar-based counts are represented as grey dots (n = 3013) and have a 14-day running mean fitted (grey line). Ground-based estimates from human observers are divided into fly-out (white dots, n = 90) and static (black dots = 156) counts^1,24^.

Radar counts exhibited a significant seasonal pattern within years (deviance = 45.4, df = 12, p < 0.001; Supplementary Fig. 1), with the number of bats in the Yarra Bend colony being highest in between February and April and lowest between July and August, and largely matching those of ground-based estimates from human observers (Fig. 1; see also below). Overall estimated flying-fox numbers were at their lowest in winter 2009 (with an estimated minimum of 10 ± 7 SE bats in May), and colony numbers peaked in January 2014 at 46,169 ± 3,097).

### Validating population estimates against colony count data

**Figure 2:**
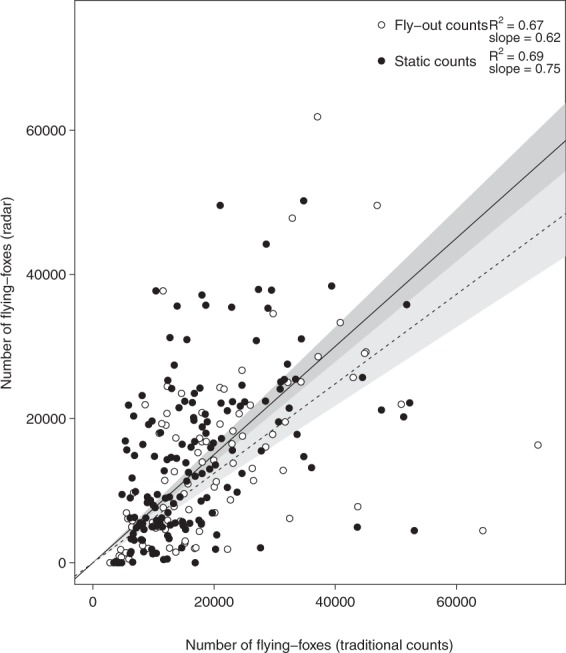
Radar-based estimates versus ground-based estimates of the numbers of flying-foxes present at the Yarra Bend roost. Ground-based counts are divided into fly-out (white dots, dashed line, n = 90) and static (black dots, solid line, n = 156) colony counts. The lines are fits from linear models constrained to pass through 0. Alpha shading bands indicating the 95% confidence intervals are included for each line as grey polygons (static counts = dark grey; fly-out counts = light grey). Adjusted R^2^s and slopes are included in the legend.

Radar estimates of flying-fox numbers at Yarra Bend were significantly positively associated with both fly-out (adjusted R^2^ = 0.67, estimate = 0.62, t = 13.63, *p* < 0.001; Fig. 2) and static counts (adjusted R^2^ = 0.69, estimate = 0.75, t = 18.58, *p* < 0.001; Fig. 2).

### Timing of emergence

**Figure 3:**
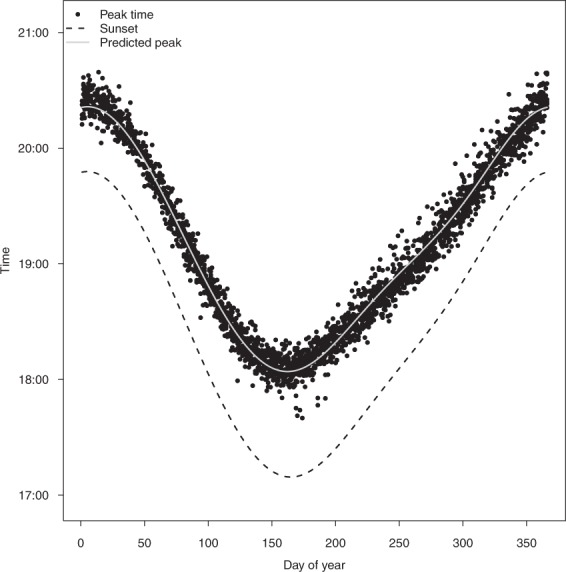
Time (AEST) of peak of flying-fox emergence (black dots, n = 2950), as determined form radar data, versus time of year at the Yarra Bend roost. Local sunset time is marked as a black dashed line and the predicted peak emergence time from a sine/cosine model is shown as a grey line.

The timing of peak emergence varied significantly relative to sunset and day of the year (adjusted R^2^ = 0.63, F_2,2947_ = 2501, *p* < 0.001; Fig. 3). Predictions from a significant sine-cosine relationship estimated emergence to range between a minimum of 34 minutes after sunset from December 26th to January 7th to a maximum of 55 minutes after sunset from 13th June to 21st July.

### Direction of departure

**Figure 4:**
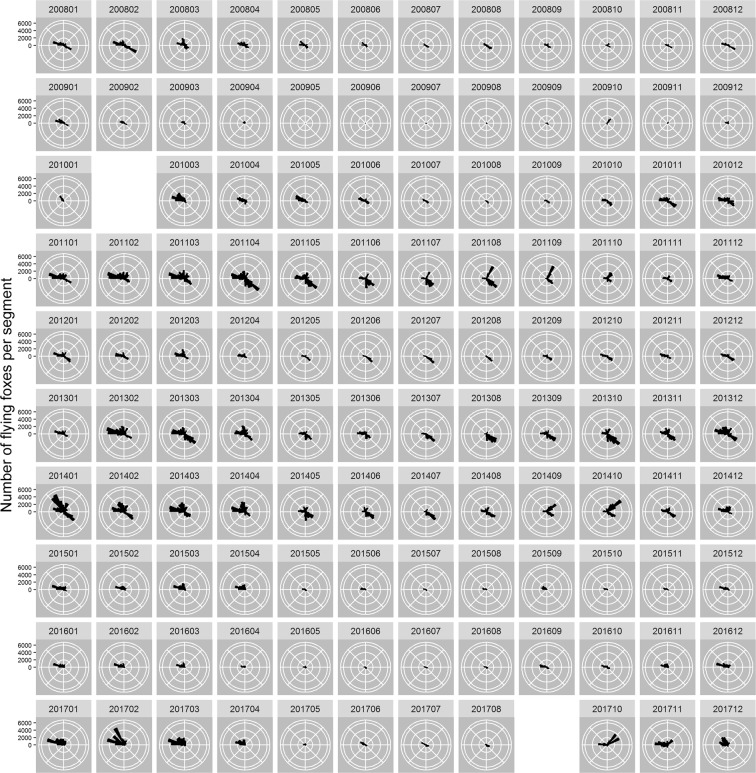
The mean numbers of flying-foxes detected by radar flying out in different directions from the Yarra Bend roost for each month of each year.

**Figure 5:**
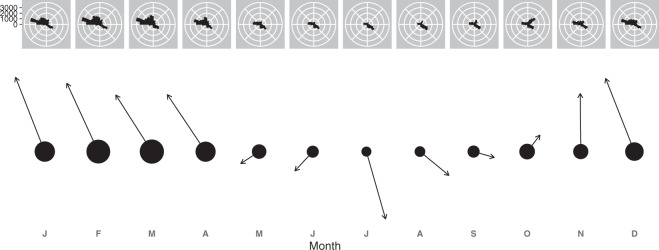
Panel 1: The mean number of flying-foxes detected by radar flying out from the Yarra Bend roost in different directions for each month across all years. Panel 2: The mean monthly departure directions. Data were averaged within each month and then for each month across all years.

There was considerable variation among months in the directions in which individuals departed the roost (Fig. 4). On average, animals were significantly oriented (Rao’s spacing tests^28^, all U > 318.8, all *p* = 0; Fig. 4) and the mean monthly directions tended to be towards the north from October to April and towards the south from April to September (Supplementary Table S1; Fig. 5).

### Extreme heat events

**Figure 6:**
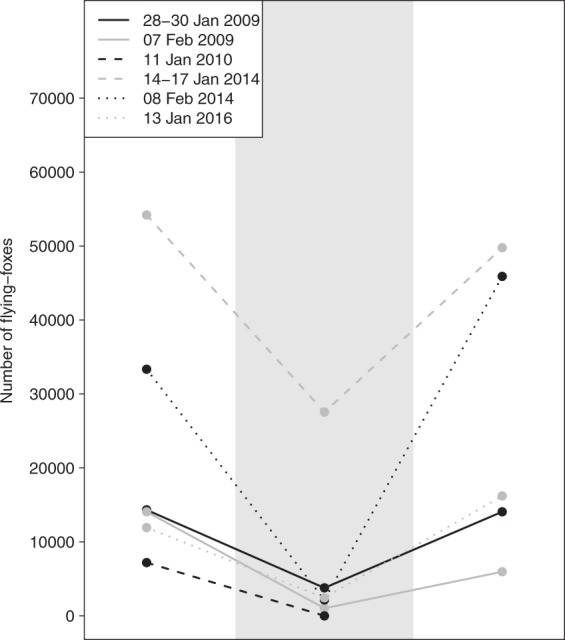
Mean numbers of flying-foxes that were detected by radar emerging in the days before, during, and after extreme heat events (T ≥ 42 °C^19^). Means were taken from the week prior to an event, the ≥1 consecutive day(s) of the event, and from the week following the event. Mean emergences (dots) are linked by individually identifiable lines for each extreme heat event. The grey polygon indicates the period of extreme heat. The date of each event is indicated by the legend.

We found that the mean number of flying-foxes emerging during an extreme heat event was significantly lower than in the week preceding, or in the week after the event (overall model: *X*^2^ = 12.33, d.f. = 5, *p* = 0.002; before *vs* during: *p* = 0.006; during *vs* after: *p* = 0.005). We found no significant difference between the mean numbers of flying-foxes emerging from the colony before and after the extreme heat event (before *vs* after: *p* = 0.693). There were decreases in the mean peak emergence in the week after an extreme heat event on 07/02/2009 (decrease of 58%), 14–17/01/2014 (decrease of 8%) and 28–30/01/2009 (decrease of 2%). Emergence increased by 36 and 38% during the week after 13/01/2016 and 08/02/2014 respectively (Fig. 6). There were no data following 11/01/2010 as the radar stopped functioning in the evening of this day.

## Discussion

Our analyses demonstrate that radar data can reliably be used to assess flying-fox colony size, as well as the timing and departure directions of emerging individuals. As such, radar represents an extremely promising tool for the long-term monitoring of vulnerable flying-fox populations at a landscape scale.

Radar population estimates largely matched those of ground-based estimates from human observers (Figs 1; 2), revealing the expected seasonal variation in colony size (largest from January-April, smallest from July-August) that has been reported for this colony based on traditional ground-based census techniques^29^. The seasonal pattern also matches those of roosts further north, with the largest aggregations occurring during the mating season in autumn^30,31^. Traditional census techniques are logistically difficult and therefore tend to be conducted infrequently, and are subject to a range of biases, especially when roosts are difficult to access or lack clear lines of sight^1,25,26^. Radar, on the other hand, can provide daily colony size estimates in near real-time, greatly enhancing the temporal resolution and immediacy of the estimates, although it suffers from its own sources of error (discussed below). The relationship between the radar estimates and both traditional count methods had a slope of less than one (fly-out: 0.62, static: 0.75, Fig. 2), which suggests that either the ground-based counts over-count or the radar estimates under-count the true size of the colony. For more accurate estimates of absolute colony size, as opposed to estimates used for relative colony size trends, we suggest that both methods would need external calibration, e.g. with absolute counts from thermal imagery from drones or helikites^32^. Nevertheless, our radar estimates matched the ground-based estimates surprisingly well (fly-out counts: R^2^ = 0.67; static counts: and 0.69; Fig. 2) indicating that radar data can provide a cost-effective, near real-time alternative to the estimates derived from traditional census techniques.

As shown here, radar-based colony size estimates can be made retrospectively, meaning we can examine the radar data for signals of important ecological events. For example, a large Spotted Gum flowering event occurred on the NSW south coast in April and May 2016^33^. The May 2016 National Flying-Fox Monitoring Program survey found that over 40% (277,000) of the entire grey-headed flying-fox population were roosting within 20 km of Batemans Bay^34^. This is matched by a dip in the numbers detected in Yarra Bend.

Another ecological event picked up by radar data is that of days of extreme heat. On all six of the extreme heat events where data were available, peak emergence numbers on days of extreme heat were significantly lower than the mean peak emergence numbers in the week preceding, or the week after the extreme heat event. No marked emergence was observed on these days even when all scans from four hours before and after sunset were examined. This suggests that fly-out behaviour is strongly affected by these extreme heat events, as has been observed anecdotally (J. Welbergen; pers. obs.).

As expected from earlier studies on flying-foxes, the radar data revealed a clear and predictable relationship between the timing of flying-fox emergence and sunset (Fig. 3). Timing of flying-fox emergence is advanced when energetic demands are highest^35,36^; therefore, emergence times from radar data can potentially be used to examine the drivers and/or correlates of flying-fox nutritional status. Furthermore, flying-fox emergence timing likely depends on ambient light levels^35,37^, and therefore radar data can provide a means to study retrospectively the impacts of anthropogenic light pollution on flying-fox emergence dynamics. Finally, whilst not examined here, radar data also have the potential to uncover information on the early morning ‘fly-in’ of flying-foxes. This could be used to examine differences in timing of fly-in associated with breeding status^38^, and could also be used as a way of validating the previous or subsequent evening’s emergence counts.

There was a clear seasonal pattern in the directions with which individuals departed the roost, with individuals predominantly flying north-westwards in summer and more or less southwards during the winter months (Fig. 5). Despite these dominant directions, there was still considerable variation in the monthly fly-out directions between years, and future work will involve associating this information with local flowering and fruiting events (e.g.^39,40^) based on direct observations and/or remote sensing (e.g.^41–43^).

The radar information has direct implications for management practice. Data from the Melbourne radar have been archived since 1998, which means that they can potentially be used to examine retrospectively the impact of the forced dispersal of the Melbourne Botanic Garden colony in 2003^44^ at a daily resolution. Likewise, this same approach can be used to monitor the impacts of flying-fox management actions, including forced dispersal of flying-foxes from their roosts^44,45^ in near real-time, elsewhere. Further, Westcott *et al*.^1,46^ identified roosting away from known roosts as the major contributor to uncertainty around current flying-fox population trend estimates. Future work could include automatic detection by radar of unknown flying-fox colonies based on the ‘signature’ of reflectivity and velocity^47,48^, and so help improve the accuracy of existing monitoring programs.

In addition, as emergence timing provides information on flying-fox nutritional status^12,35^ this means that radar data can provide an early warning of food shortages that are associated with low postpartum female weight^49^, reduced reproductive success^50^, and with flying-fox damage to commercial fruit crops^51^. Furthermore, the lack of significant emergence signals on days when colonies experience extreme heat can also provide a proxy as to the potential impact on the colony, and it is possible that the difference in mean peak emergence size before and after an extreme heat event can be related to flying-fox die-off numbers^19^.

The radar information also has applications outside the ecological realm. Flying-fox strikes are a serious hazard to both commercial and military aircraft^52^, and radar-derived data on the quantity, timing, and direction of emerging flying-foxes can be used to inform the scheduling of aircraft take-off and landing. For example, Melbourne Airport is 20.4 km almost due northwest (310°) of the Yarra Bend colony. This is a common departure direction from the colony, with substantial proportions of individuals departing in this direction at most times of the year (Fig. 5). As our data suggest, the relatively highest risk of aircraft strike in Melbourne would be between 19:15 and 20:30 AEST from December - March (Supplementary Table S2). Radar may also be used to inform risk of flying-fox strike at windfarms, potentially improving the macro- and micro-siting of windfarms and turbines, as well as informing the shut-down of turbines to prevent strike of flying-foxes.

Whilst radar data can provide a wealth of ecological information and have important practical applications, they are limited by the distance between radar and roost site, and by terrain. Records from plane strikes suggest that the majority of flying-foxes fly at *c*. 150 m, with 400 m likely to be the upper limit of flight height^53^. This means that, if flying-foxes are flying at an altitude of 400 m, the maximum distance that colonies can be detected by the lowest scan of a weather radar (typically 0.5°) is *c*. 70 km from the position of the radar (though the elevation of the radar and the colony must also be taken into account). Terrain can pose obstacles to the detection of emerging flying-foxes, e.g. when a mountain is between the radar and a flying-fox colony. Without taking topography into account and based only on distance from the radar, we find that 324 of the 545 colonies (59%) currently monitored as part of the national flying-fox monitoring program are within 70 km of a radar for which data is available (see Table S3), and thus may be appropriate for radar monitoring.

Another limitation is that, in order to obtain a clean meteorological-radar product, some on-site processing occurs at the radar site before data are transmitted elsewhere^4,54^, frequently filtering out the aeroecological signals of interest before the data are made available to ecologists (e.g., this is the case for the archived Sydney radar data). It is thus important that unfiltered data be stored. In addition, on some days, large weather systems can completely obscure emerging flying-foxes and results in severe colony size overestimates, although in our case such days are always filtered out using the MAD filtering procedure (see methods). On other days, smaller weather systems overlap with flying-fox emergence and when these days are included in the flying-fox estimates this can potentially lead to colony size overestimates. Finally, clutter filtering processes can be inaccurate, resulting in false-positive and/or false-negative detections of flying-foxes, introducing errors in the population estimates.

Some of the above limitations can be addressed by the recent (2018) upgrade of the Australian weather radar network to dual-polarisation at stations in Adelaide, Melbourne, Sydney and Brisbane^55^. Dual-polarisation radars make measurements at two orthogonal polarizations, one aligned horizontally and one aligned vertically, to obtain additional information on the shape, size, orientation, and behaviour of objects in the airspace^56^. These dual-polarisation radars hold the promise of expanding and enhancing the monitoring techniques described here, while enabling entirely new ecological applications such as automated data processing algorithms to facilitate large-scale and long-term analyses in biogeography, phenology, and movement ecology of flying-foxes and other volant organisms in Australia.

## Methods

### Radar data

Ten years of historical radar observations spanning 03/01/2008-31/12/2017 were obtained for the weather surveillance radar in Melbourne (location: 37.852°S, 144.752°E) from the Australian Bureau of Meteorology (BOM) in the HDF5 file format^57^. Each formatted radar data file is a single volume scan of the airspace conducted over a 5–10 min interval and is comprised of a series of single-elevation sweeps. Each sweep is a single rotation of the radar antenna in azimuth and results in a two-dimensional array of voxels having dimensions in azimuth (360 rays at 1-degree resolution) and range (600 or 896 bins at 250-metre resolution). Depending on the scan type, the maximum range covered is either 150 km or 224 km from the radar site. Sweeps are made at the 0.5-, 0.9-, 1.3-, 1.8-, 2.4-, 3.1-, 4.2-, 5.6-, 7.4-, 10.0-, 13.3-. 17.9-, 23.9- and 32.0-degree elevation angles. The archived radar products include raw (i.e., unfiltered) horizontally-polarized reflectivity factor (Z_h_) in units of dBZ and radial velocity (V_rad,h_) in m/s^57^. The Yarra Bend colony is 24.2 km from the radar, thus the lowest volume of air scanned in the lowest three scans is at 46, 215 and 384 m above colony height (taking into account the height of the radar antenna of 42 m above sea level and a colony elevation of 30 m^58^).

### Radar data processing

The radar reflectivity factor (Z_h_) of each voxel was converted to reflectivity (η) following Chilson *et al*.^36^. The absolute number of flying-foxes in each voxel was estimated by dividing reflectivity (cm^2^/km^3^) by the estimated radar cross section (RCS) of a single flying-fox (cm^2^), and multiplying the result by the volume of each voxel (km^3^). The RCS of a grey-headed flying-fox was estimated at C-band using the electromagnetic modelling procedure outlined in^59^. An ‘average’ grey-headed flying-fox model was obtained by re-scaling an existing *Tadarida brasiliensis* model to a wingspan of 100 cm (J. Welbergen pers. comm.). The volume of individual sampling voxels was calculated from beam geometry, assuming a one degree rotationally symmetric circular conical frustum. Next, a velocity filter was applied, where only voxels with a velocity in the range of −17 to 17 m/s (negative values indicate movement towards the radar and positive values indicate movement away from the radar) were retained. This encompasses the range of speeds that flying-foxes are likely to fly^60^. Voxels containing <1.5 flying-fox/km^3^ were set to zero in order to create ‘patches’ of voxels containing aerial matter. Such signatures occurring at dusk over known bat colony sites are readily associated with flying-fox emergences, and these patterns are not reproduced by birds or insects. These ‘patches’ of aerial matter were retained if they fulfilled two criteria: patch edges had to be within 5 km of the colony location, and patches had to be larger than 0.3 km^2^. These criteria were based on visual examination of the emerging patches of flying-foxes in Z_h_. Finally, a probability density function was used to plot the putative number of flying-foxes per voxel. This usually created a bimodal histogram where the first peak is that of flying-foxes and the second is meteorological signals or clutter. The lowest point between these two peaks was selected as the cut-off point below which all remaining voxels are presumed to contain radar signals originating from flying-foxes, and the remaining voxels are removed. The total estimate of flying-foxes aloft per scan was obtained by summing flying-fox numbers across voxels within a 50 km radius of the colony for the three lowest radar scans (centre of beam = 0.5°, 0.9°, 1.3°). While some birds and insects are likely present in the airspace above the colony site, their small body sizes and low concentrations relative to the emerging flying-foxes are assumed to contribute negligibly to the final estimates. All data processing and visualisation of radar data was done in Python using the ‘wradlib’, ‘matplotlib’ and ‘numpy’ packages.

### Long term flying-fox counts

All available data from the Melbourne radar from January 2008 to December 2017 were processed. There were 222 days of missing data due to files not being saved before deletion, to the radar breaking down or being taken offline for maintenance during this time period, notably from 12/01/2010-03/03/2010 and 26/08/2017-10/10/2017 as well as other relatively shorter periods of time. The maximum number of flying-foxes that were simultaneously aloft each day (hereafter referred to as peak emergence) was determined. On some days a significant weather system coincided with flying-fox emergence, resulting in a patch of precipitation over the colony site. These cases were infrequent and produced anomalously large values of peak emergence. As a result, a simple outlier analysis on peak emergence could successfully identify these cases. To quantify the characteristic annual variability in colony size, the median absolute deviation (MAD^61^) of peak emergence was calculated for each year. Values greater than three MAD above the median were indicative of outlier cases associated with weather signals and removed from the analysis. After filtering, 3013 of 3431 (88%) of observations remained. We used the ‘ets’ function of the R package forecast^62^ to detect whether seasonality was present among monthly radar counts means (N = 120 months).

### Validating population estimates against colony count data

Flying-fox numbers at the Yarra Bend colony in greater Melbourne (37.7831° S, 145.0290° E) were counted *c*. fortnightly to monthly from 2003–2018 (on-going). From 1986–2003, flying-foxes occupied the Royal Botanic Gardens Melbourne, and in 2003 this roost was forcibly dispersed with a new site established in Yarra Bend Park^24^. Ground-based counts were carried out in two ways: fly-out counts were performed once per month by 10–20 volunteers with a range of experience^29^, and static counts were conducted once to twice per month by a small number of experienced researchers. The colony was counted on 281 days from 2008–2017, radar data were available on 261 of these days, and 230 days where both radar and ground-based count data were available remained following filtering based on MAD (see above). These radar estimates of flying-fox numbers were compared to both fly-out and static counts separately using a linear model constrained to pass through 0 (fly-out: n = 90; static: n = 156). On 16 days the colony was counted on the same day using both traditional methods.

### Timing of emergence

Timing of peak emergence was established for each day that reliable radar data were available, and for days when the fly-out estimate was greater than 0 (n = 2950). A sine-cosine equation was used to establish a predictive relationship between peak emergence relative to sunset and the day of the year. Sunset times at Yarra Bend were calculated using the R package ‘suncalc’.

### Direction of departure

To examine the direction of flying-fox departures from the Yarra Bend roost, the 50 km radius surrounding the colony was divided into 32 equal-sized segments and the number of bats in each segment during peak emergence was calculated for each day. These data were averaged over each month and plotted in circular histograms. The mean departure direction^28^ was calculated for each month, and Rao’s spacing test^28^ was used to determine the ‘directionality’ of departures for each month.

### Extreme heat events

There were seven extreme heat events in Melbourne where temperatures exceeded the critical threshold of 42 °C^19^ for ≥1 day during 2008–2017 (Bundoora weather station, BOM climate data online). Five of these events were individual days, and the remaining days fell in two clusters of three and four days (28–30/01/2009; 07/02/2009; 11/01/2010; 14–17/01/2014; 08/02/2014; 19/12/2015; 13/01/2016). Radar counts were available for at least one day for six of these extreme heat events (on 19/12/2015 flying-fox emergence was obscured by meteorological signals). The mean emergence numbers were calculated for the week preceding the first day of an extreme heat event, and for the week following the last day of the extreme heat event. A linear mixed effects model was constructed with mean emergence as the independent variable, and a categorical variable of before, during and after an extreme heat event as the explanatory variable. Event was included as a random factor.

## Supplementary information

Supplementary Information

## Data Availability

Radar data are available for download from http://openradar.io/. The list of radar locations was downloaded from: http://dapds00.nci.org.au/thredds/catalog/rq0/odim_pvol/catalog.html?dataset=rq0/odim_pvol/radar_site_list.csv.
