# Supplementary material for: Using weather radar to monitor the number, timing and directions of flying-foxes emerging from their roosts
Source: Sci Rep. 2019 Jul 15;9:10222. doi: 10.1038/s41598-019-46549-2 (PMC6629676; doi:10.1038/s41598-019-46549-2)

# Supplementary Information

## Using weather radar to monitor the number, timing and directions of flying-foxes emerging from their roosts

Jessica Meade, Rodney van der Ree, Phillip M. Stepanian, David Westcott, Justin A. Welbergen

**Supplementary Table S1.**

| Date | Rao Statistic | Rao P-value | Mean direction (°) | Mean colony size |
|---|---|---|---|---|
| 200801 | 359.4 | 0 | 337 | 17952 |
| 200802 | 359.5 | 0 | 346 | 23648 |
| 200803 | 359.5 | 0 | 356 | 21513 |
| 200804 | 359.3 | 0 | 330 | 17120 |
| 200805 | 359.1 | 0 | 290 | 13047 |
| 200806 | 358.6 | 0 | 316 | 8054 |
| 200807 | 358.3 | 0 | 165 | 6972 |
| 200808 | 358.9 | 0 | 112 | 9372 |
| 200809 | 358.5 | 0 | 46 | 7678 |
| 200810 | 358.1 | 0 | 67 | 5976 |
| 200811 | 358.3 | 0 | 57 | 6978 |
| 200812 | 359.0 | 0 | 352 | 11059 |
| 200901 | 359.2 | 0 | 317 | 12568 |
| 200902 | 358.6 | 0 | 320 | 8125 |
| 200903 | 358.3 | 0 | 298 | 6933 |
| 200904 | 357.0 | 0 | 321 | 3903 |
| 200905 | 258.8 | 0 | 266 | 10 |
| 200906 | 347.0 | 0 | 245 | 367 |
| 200907 | 353.6 | 0 | 93 | 1365 |
| 200908 | 353.8 | 0 | 30 | 1814 |
| 200909 | 356.0 | 0 | 121 | 2831 |
| 200910 | 357.9 | 0 | 38 | 4605 |

| | | | | |
|---|---|---|---|---|
| 200911 | 354.7 | 0 | 36 | 2122 |
| 200912 | 358.3 | 0 | 327 | 6220 |
| 201001 | 358.4 | 0 | 309 | 6223 |
| 201003 | 359.5 | 0 | 308 | 22537 |
| 201004 | 359.3 | 0 | 284 | 17513 |
| 201005 | 359.3 | 0 | 290 | 17628 |
| 201006 | 359.3 | 0 | 287 | 15022 |
| 201007 | 358.5 | 0 | 179 | 7782 |
| 201008 | 357.4 | 0 | 143 | 4243 |
| 201009 | 358.4 | 0 | 131 | 7288 |
| 201010 | 359.2 | 0 | 194 | 13837 |
| 201011 | 359.5 | 0 | 349 | 23119 |
| 201012 | 359.5 | 0 | 345 | 25038 |
| 201101 | 359.6 | 0 | 322 | 26768 |
| 201102 | 359.7 | 0 | 333 | 34342 |
| 201103 | 359.7 | 0 | 334 | 38555 |
| 201104 | 359.7 | 0 | 280 | 45686 |
| 201105 | 359.7 | 0 | 115 | 35709 |
| 201106 | 359.5 | 0 | 159 | 24204 |
| 201107 | 359.5 | 0 | 137 | 21449 |
| 201108 | 359.5 | 0 | 103 | 22439 |
| 201109 | 359.3 | 0 | 73 | 15194 |
| 201110 | 359.4 | 0 | 54 | 18427 |
| 201111 | 359.2 | 0 | 55 | 13636 |
| 201112 | 359.4 | 0 | 312 | 20154 |
| 201201 | 359.4 | 0 | 335 | 20322 |
| 201202 | 359.3 | 0 | 282 | 16884 |
| 201203 | 359.4 | 0 | 310 | 19370 |
| 201204 | 359.1 | 0 | 291 | 12745 |
| 201205 | 358.7 | 0 | 191 | 8821 |
| 201206 | 358.5 | 0 | 141 | 7752 |

| | | | | |
|---|---|---|---|---|
| 201207 | 358.7 | 0 | 140 | 8612 |
| 201208 | 358.1 | 0 | 131 | 6018 |
| 201209 | 358.9 | 0 | 162 | 10345 |
| 201210 | 359.1 | 0 | 202 | 12573 |
| 201211 | 359.1 | 0 | 275 | 12323 |
| 201212 | 359.3 | 0 | 305 | 15713 |
| 201301 | 359.3 | 0 | 322 | 16020 |
| 201302 | 359.7 | 0 | 312 | 34741 |
| 201303 | 359.7 | 0 | 256 | 38184 |
| 201304 | 359.6 | 0 | 339 | 31571 |
| 201305 | 359.4 | 0 | 184 | 19435 |
| 201306 | 359.4 | 0 | 254 | 19468 |
| 201307 | 359.3 | 0 | 155 | 15730 |
| 201308 | 359.4 | 0 | 136 | 19242 |
| 201309 | 359.5 | 0 | 130 | 23133 |
| 201310 | 359.6 | 0 | 129 | 30539 |
| 201311 | 359.6 | 0 | 31 | 27422 |
| 201312 | 359.7 | 0 | 335 | 36184 |
| 201401 | 359.8 | 0 | 351 | 46169 |
| 201402 | 359.7 | 0 | 337 | 35854 |
| 201403 | 359.7 | 0 | 311 | 37493 |
| 201404 | 359.7 | 0 | 322 | 33855 |
| 201405 | 359.6 | 0 | 140 | 28493 |
| 201406 | 359.5 | 0 | 112 | 21616 |
| 201407 | 359.3 | 0 | 128 | 15517 |
| 201408 | 359.4 | 0 | 74 | 20671 |
| 201409 | 359.5 | 0 | 66 | 21093 |
| 201410 | 359.6 | 0 | 62 | 26159 |
| 201411 | 359.5 | 0 | 38 | 19527 |
| 201412 | 359.5 | 0 | 337 | 21483 |
| 201501 | 359.4 | 0 | 329 | 18783 |

| | | | | |
|---|---|---|---|---|
| 201502 | 359.2 | 0 | 332 | 14685 |
| 201503 | 359.4 | 0 | 320 | 18204 |
| 201504 | 359.3 | 0 | 317 | 15721 |
| 201505 | 358.3 | 0 | 240 | 6642 |
| 201506 | 358.0 | 0 | 284 | 5827 |
| 201507 | 357.2 | 0 | 242 | 4163 |
| 201508 | 357.8 | 0 | 301 | 5033 |
| 201509 | 358.3 | 0 | 302 | 6622 |
| 201510 | 357.6 | 0 | 294 | 4865 |
| 201511 | 357.8 | 0 | 294 | 5331 |
| 201512 | 358.9 | 0 | 314 | 10516 |
| 201601 | 359.1 | 0 | 318 | 13047 |
| 201602 | 359.0 | 0 | 310 | 12049 |
| 201603 | 359.0 | 0 | 316 | 11486 |
| 201604 | 358.2 | 0 | 313 | 6384 |
| 201605 | 357.9 | 0 | 326 | 5530 |
| 201606 | 357.5 | 0 | 171 | 4698 |
| 201607 | 357.4 | 0 | 155 | 4438 |
| 201608 | 357.9 | 0 | 182 | 5391 |
| 201609 | 358.9 | 0 | 285 | 10135 |
| 201610 | 358.9 | 0 | 325 | 10160 |
| 201611 | 359.1 | 0 | 330 | 12665 |
| 201612 | 359.2 | 0 | 325 | 15311 |
| 201701 | 359.5 | 0 | 325 | 25095 |
| 201702 | 359.7 | 0 | 323 | 34893 |
| 201703 | 359.6 | 0 | 314 | 29048 |
| 201704 | 359.3 | 0 | 314 | 16170 |
| 201705 | 357.9 | 0 | 336 | 5627 |
| 201706 | 358.6 | 0 | 296 | 8090 |
| 201707 | 358.5 | 0 | 168 | 7481 |
| 201708 | 358.3 | 0 | 119 | 6630 |

| | | | | |
|---|---|---|---|---|
| 201710 | 359.6 | 0 | 29 | 26100 |
| 201711 | 359.5 | 0 | 298 | 25097 |
| 201712 | 359.5 | 0 | 310 | 21761 |

**Supplementary Table S1.** This shows the Rao statistic, Rao p-value, mean departure direction and mean colony size, for each month that radar data is available from January 2008 to December 2017.

**Supplementary Table S2.**

| Month | Time (AEST) | Number of flying-foxes | Mean emergence distance (km) | Maximum emergence distance (km) | Relative strike risk | Risk rating |
|---|---|---|---|---|---|---|
| Jan | 20:21 | 1332 | 10.6 | 24.0 | 0.85 | High |
| Feb | 20:00 | 1516 | 10.0 | 32.6 | 1.00 | High |
| Mar | 19:19 | 1483 | 9.4 | 25.7 | 0.97 | High |
| Apr | 18:41 | 1125 | 8.4 | 19.0 | 0.68 | Moderate |
| May | 18:12 | 653 | 5.9 | 20.2 | 0.29 | Low |
| Jun | 18:06 | 544 | 5.3 | 31.0 | 0.20 | Low |
| Jul | 18:19 | 295 | 3.9 | 17.0 | 0.00 | Low |
| Aug | 18:38 | 318 | 3.8 | 17.4 | 0.02 | Low |
| Sep | 18:58 | 558 | 5.6 | 15.9 | 0.22 | Low |
| Oct | 19:23 | 447 | 6.1 | 16.2 | 0.12 | Low |
| Nov | 19:49 | 772 | 7.7 | 19.8 | 0.39 | Moderate |
| Dec | 20:14 | 1206 | 9.4 | 29.8 | 0.75 | High |

**Supplementary Table 2.** The monthly mean number of flying-foxes departing in the direction of Melbourne airport is shown. This is based on a segment centred on 309.4° (the direction of Melbourne airport from the colony is 310°). Mean time of peak emergence is also shown. Relative strike risk is the number of flying-foxes normalised from 0-1 such that 1 is the maximum number of flying-foxes and 0 is the minimum number.

**Supplementary Table S3.**

| BOM ID | Short name | Site latitude | Site longitude | State | Number of NFFMP colonies closer than 70 km |
|---|---|---|---|---|---|
| 1 | CampRd | -37.691 | 144.946 | VIC | 3 |
| 2 | Melb | -37.8553 | 144.7554 | VIC | 4 |
| 3 | Wollgng | -34.2624 | 150.8751 | NSW | 30 |
| 4 | LemnTre | -32.7298 | 152.0254 | NSW | 27 |
| 8 | Kanign | -25.9574 | 152.5768 | QLD | 16 |
| 11 | Adel | -34.95 | 138.533 | SA | 1 |
| 19 | Cairns | -16.8182 | 145.6629 | QLD | 43 |
| 20 | Twnsvl | -19.25 | 146.767 | QLD | 2 |
| 21 | MtStrt | -19.35 | 146.783 | QLD | 2 |
| 22 | Mackay | -21.1172 | 149.2172 | QLD | 10 |
| 23 | Gladstn | -23.8551 | 151.2626 | QLD | 6 |
| 24 | Bowen | -19.8857 | 148.0757 | QLD | 3 |
| 28 | Grafton | -29.6207 | 152.9634 | QLD | 20 |
| 34 | CairnAP | -16.873 | 145.746 | QLD | 41 |
| 35 | CoffsH | -30.317 | 153.117 | NSW | 16 |
| 40 | CapFlat | -35.6614 | 149.5122 | ACT | 7 |
| 42 | Tindal | -14.5109 | 132.447 | NT | 3 |
| 43 | BrisAP | -27.392 | 153.13 | QLD | 75 |
| 46 | Sellick | -35.3296 | 138.5025 | SA | 1 |

| | | | | | |
|---|---|---|---|---|---|
| 47 | Rhmptn | -23.383 | 150.467 | QLD | 4 |
| 49 | NEVic | -36.0296 | 146.0228 | VIC | 1 |
| 50 | Marburg | -27.6064 | 152.5401 | QLD | 55 |
| 51 | MelbnAP | -37.6656 | 144.8312 | VIC | 4 |
| 54 | Kurnell | -34.0148 | 151.2262 | NSW | 27 |
| 57 | Esale | -38.12 | 147.13 | VIC | 6 |
| 64 | BuckPk | -34.617 | 138.4689 | SA | 1 |
| 66 | MtStapl | -27.7178 | 153.24 | QLD | 102 |
| 68 | Bnsdale | -37.8876 | 147.5755 | VIC | 6 |
| 71 | THills | -33.7008 | 151.2095 | NSW | 26 |
| 72 | Emerald | -23.5496 | 148.2392 | QLD | 1 |
| 73 | HrvyRng | -19.4199 | 146.5509 | QLD | 2 |
| 75 | MntIsa | -20.7112 | 139.5553 | QLD | 1 |

**Supplementary Table S3.** A list of radars (from: http://dapds00.nci.org.au/thredds/catalog/rq0/odim_pvol/catalog.html?dataset=rq0/odim_pvol/radar_site_list.csv) that are within 70 km of flying-fox colonies monitored by the National Flying-Fox Monitoring Program since January 2017. Flying-fox camp data comes from https://www.environment.gov.au/webgis-framework/apps/ffc-wide/ffc-wide.jsf . 70 km is the maximum distance that it is likely that flying-foxes could be detected by the radar without taking topography into account.

**Supplementary Figure S1.** A box-and-whisker plot showing the median (middle line) monthly colony count from radar data across years. Also shown is the interquartile range (IQR, box) and the minimum/maximum values 1.5×IQR (whiskers).

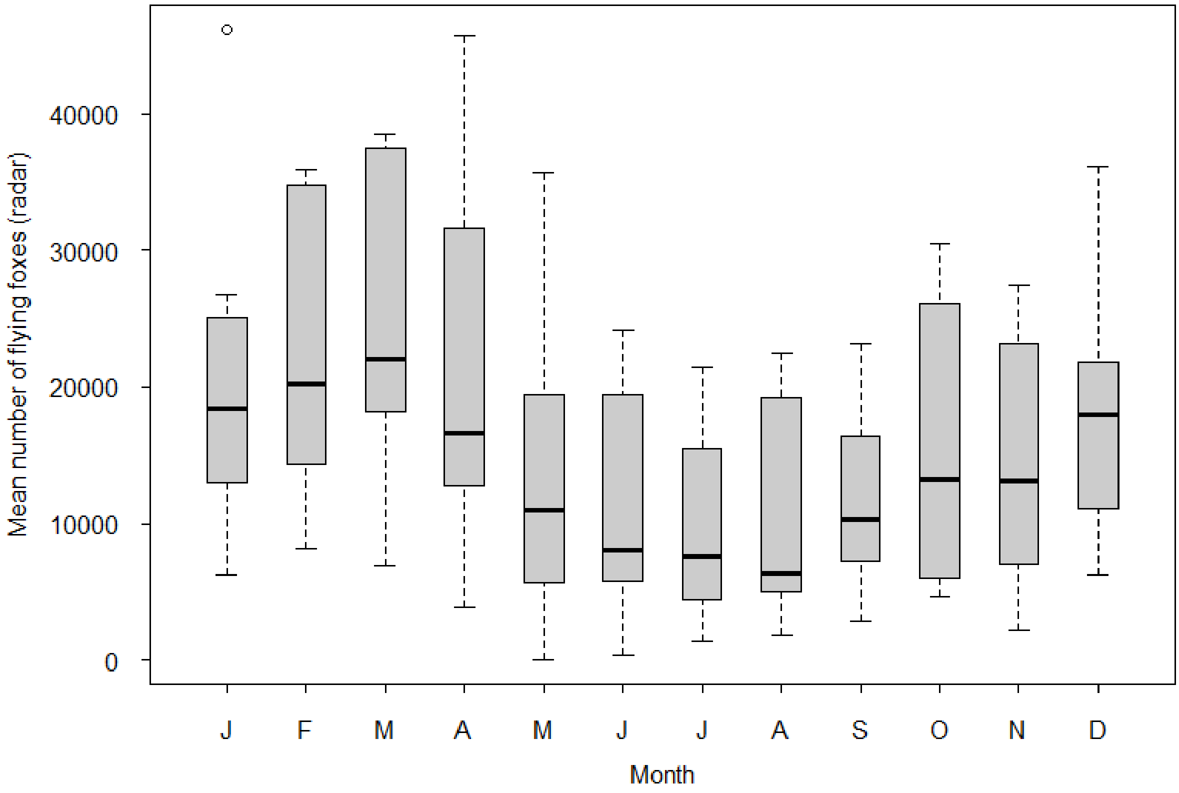

Supplement: Supplementary file 1 — Supplementary Information [file 41598_2019_46549_MOESM1_ESM.pdf]
